# Towards a Sustainable Vector-Control Strategy in the Post Kala-Azar Elimination Era

**DOI:** 10.3389/fcimb.2021.641632

**Published:** 2021-03-09

**Authors:** Rajesh Garlapati, Eva Iniguez, Tiago D. Serafim, Prabhas K. Mishra, Basab Rooj, Bikas Sinha, Jesus G. Valenzuela, Sridhar Srikantiah, Caryn Bern, Shaden Kamhawi

**Affiliations:** ^1^ Bihar Technical Support Program, CARE India Solutions for Sustainable Development, Patna, India; ^2^ Vector Molecular Biology Section, Laboratory of Malaria and Vector Research, National Institute of Allergy and Infectious Diseases, National Institutes of Health, Rockville, MD, United States; ^3^ Department of Epidemiology and Biostatistics, University of California, San Francisco, CA, United States

**Keywords:** visceral leishmaniasis, Kala-azar, *Phlebotomus argentipes*, vector control, Bihar, India, elimination

## Abstract

Visceral leishmaniasis (VL) is a potentially deadly parasitic disease. In the Indian sub-continent, VL is caused by *Leishmania donovani* and transmitted *via* the bite of an infected *Phlebotomus argentipes* female sand fly, the only competent vector species in the region. The highest disease burden is in the northern part of the Indian sub-continent, especially in the state of Bihar. India, Bangladesh, and Nepal embarked on an initiative, coordinated by World Health Organization, to eliminate VL as a public health problem by the year 2020. The main goal is to reduce VL incidence below one case per 10,000 people through early case-detection, prompt diagnosis and treatment, and reduction of transmission using vector control measures. Indoor residual spraying, a major pillar of the elimination program, is the only vector control strategy used by the government of India. Though India is close to its VL elimination target, important aspects of vector bionomics and sand fly transmission dynamics are yet to be determined. To achieve sustained elimination and to prevent a resurgence of VL, knowledge gaps in vector biology and behavior, and the constraints they may pose to current vector control methods, need to be addressed. Herein, we discuss the successes and failures of previous and current vector-control strategies implemented to combat kala-azar in Bihar, India, and identify gaps in our understanding of vector transmission towards development of innovative tools to ensure sustained vector control in the post-elimination period.

## Introduction 

Visceral leishmaniasis (VL) caused by two species of the protozoan parasite *Leishmania*, *L. donovani* and *L. infantum*, is the most severe form of leishmaniasis. VL is transmitted by the bite of an infected female phlebotomine sand fly. In the Indian sub-continent (ISC), *L. donovani* is the causative agent and is transmitted exclusively by *Phlebotomus argentipes*; *Phlebotomus papatasi*, the only other human-biting sand fly species present in the region, lacks vectorial capacity for *L. donovani* (Singh et al., 2013). The disease is characterized by prolonged fever, anemia, and splenomegaly and nearly always results in death if no treatment is provided (Burza et al., 2018; WHO, 2020).

Elimination of VL in the ISC is considered possible because the disease has no known animal reservoir (Singh et al., 2013), and the only proven disease vector, *Ph. argentipes*, remains susceptible to pyrethroid insecticides (WHO, 2005; WHO, 2012 ;Chowdhury et al., 2016). In 2005, a collective VL elimination framework supported by the World Health Organization (WHO) was launched by the governments of India, Bangladesh, and Nepal with an initial target date of 2015, later postponed to 2020 (WHO, 2005). The goal of the current program is “elimination as a public health problem”, defined as maintaining incidence at the block level of less than one VL case per 10,000 population per year. This goal is not equivalent to “elimination” as the term is used for polio, onchocerciasis, or dracunculiasis, for example. Elimination in those programs is defined as cessation of transmission in a region, such that interventions are no longer needed after an end date defined by the transmission cycle of the pathogen (Dowdle, 1998). By contrast, the current VL elimination program in the ISC does not aim to stop transmission entirely, only to keep VL incidence below a publically accepted threshold; in the absence of interruption of transmission, some interventions may be needed in perpetuity (Lockwood et al., 2014).

In India, the VL program target was reached in 70% of endemic blocks by 2015 (WHO, 2015). Nevertheless, VL remains a major public health concern in 2020, particularly in the impoverished states of Bihar and Jharkhand that account for more than 90% of the Indian case load (Boelaert et al., 2009; National Vector Borne Disease Control Programme, 2017; National Vector Borne Disease Control Programme, 2020). The pillars of the program include systematic surveillance, early case-detection and effective treatment, and transmission reduction through vector control (National Vector Borne Disease Control Programme, 2017; Burza et al., 2018; WHO, 2020). Vector control in the ISC depends primarily on indoor residual spraying (IRS) (WHO, 2005; WHO, 2012). This strategy may not be sufficient, especially in India. Conventionally, *Ph. argentipes* has been assumed to be almost exclusively endophilic and endophagic (Chowdhury et al., 2016). However, recent reports from Bihar highlight its exophilic and exophagic behavior, suggesting that to be effective, vector control must also encompass outdoor sand fly populations, especially in places where many residents sleep outdoors (Poche et al., 2011; Poche et al., 2012; Poche DM et al., 2017; Govil et al., 2018; Poche et al., 2018b).

This review focuses on Bihar, India, its current vector control practices, and major knowledge gaps in vector biology. Understanding these gaps will be critical to achieve and sustain the VL framework target. Additionally, we will discuss the importance of developing epidemiological tools to assess the efficiency of vector control strategies, to evaluate VL risk, and to support outbreak management after the target incidence is achieved.

## Efficacy of Vector Control Efforts in Bihar, India 

### Indoor Residual Spraying 

In the 1950s and 1960s, widespread blanket IRS using Dichlorodiphenyltrichloroethane (DDT) was implemented as the principle modality of the National Malaria Eradication Programme in India. As a collateral benefit, IRS is thought to have decreased sand fly populations and to have led to the near disappearance of VL cases from Bihar state (Sanyal et al., 1979; Thakur, 2007; Ostyn et al., 2008). After DDT spraying was halted in 1964, an explosive resurgence in VL cases followed within a decade (Sen Gupta, 1975; Sanyal et al., 1979). Epidemic cycles comprising hundreds of thousands of VL cases occurred in the late 1970s and early 1990s (Courtenay et al., 2017). In response to the epidemic of the early 1990s, IRS with DDT (5%) was implemented; although direct data are lacking, the subsequent decline in VL cases was attributed to this intervention plus the wider use of sodium stibogluconate (SSG) treatment (Thakur, 2007; Ostyn et al., 2008). In the early 2000s, the third epidemic cycle since the end of the malaria eradication era began, with annual reported VL cases rising from 12,000 in 2001 to 44,000 in 2007 (Alvar et al., 2012). When the VL resurgence was recognized in the early 2000s, the same vector control strategy was adopted. Unfortunately, by this time clinical resistance to SSG had emerged (Sundar et al., 2000). From the above, it is important to emphasize that low incidence periods of 15 to 20 years follow the natural cycle of epidemics and must be taken into account when evaluating the impact of interventions (Courtenay et al., 2017). Additionally, severe underreporting of VL cases prior to the initiation of the elimination program should also be considered when examining early VL incidence data (Singh et al., 2006; Singh et al., 2010b).

Quality of insecticide application, household coverage and sand fly susceptibility can all alter the effectiveness of IRS on targeted vector populations. Several studies identified issues which could decrease the effectiveness of vector control practices in India. These include poor training of spray workers in fundamental skills such as mixing of chemicals, and limited coverage ranging from 12 to 63.9% at the household level during spraying of active foci (Huda et al., 2011; Hasker et al., 2012). As a result, DDT residue levels on walls varied from 66 to 90% of the recommended concentration at the village level and varied from 9.1 to 330% at the house level (Chowdhury et al., 2011b).

**Figure 1:**
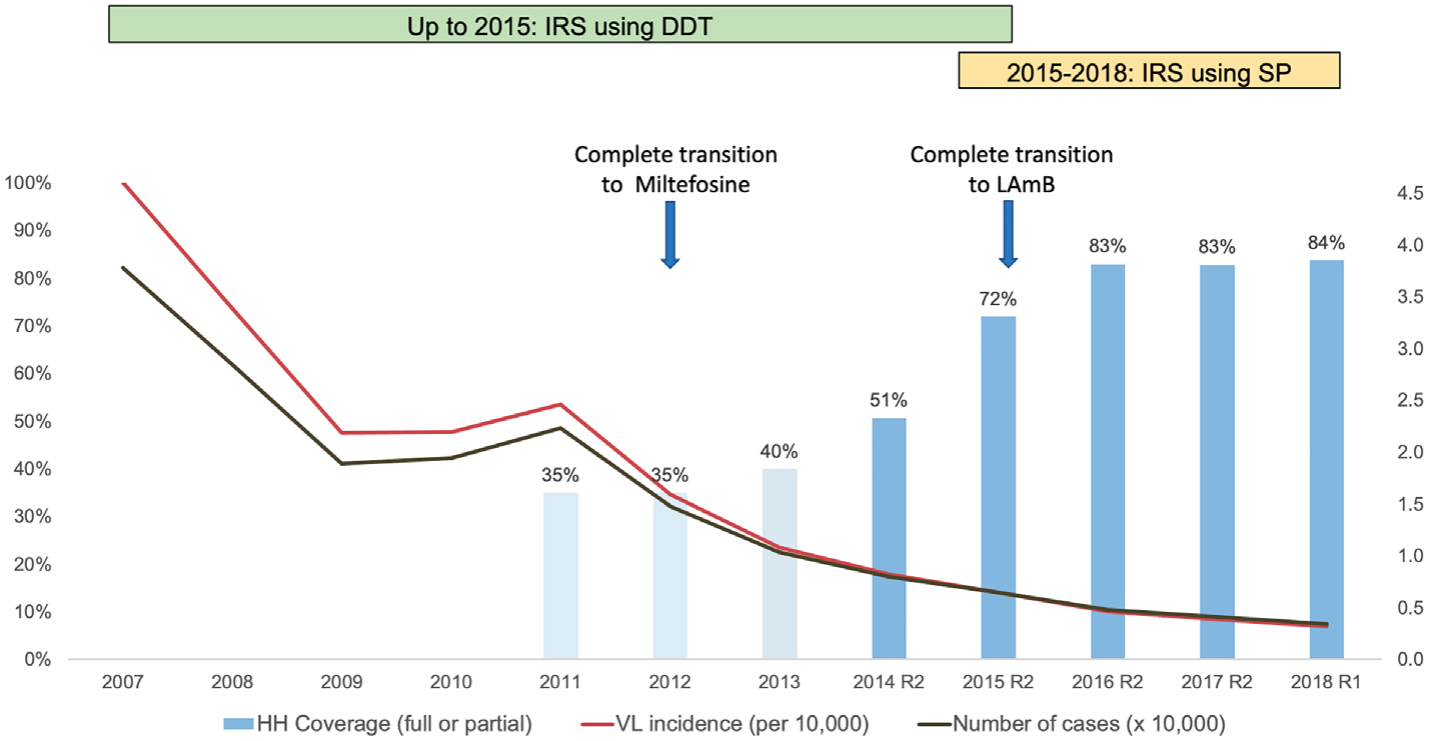
Association of indoor residual spray (IRS) coverage with visceral leishmaniasis (VL) case incidence in Bihar, 2007–2018. The incidence curve shows no sign of ‘bending’ either with increased IRS coverage or with the introduction of synthetic pyrethroids (SP) and liposomal amphotericin B (LAmB). Miltefosine, first introduced in 2008, replaced Sodium Stibogluconate as the first line of treatment by 2012. The transition from Miltefosine to LAmB began at the end of 2014 and was completed by the end of 2015. Assessment of IRS coverage, expressed as a percentage (%) of targeted households (HH) sprayed during one round of insecticide application, was conducted from 2014 onwards, through large-scale household surveys conducted by specially constituted measurement teams of CARE India, independently of program implementers, after each round of IRS. IRS coverage before 2014 (lighter shaded columns) is extrapolated from available data at 30–40%, and based on observations of all components of IRS operations during that period. R1 and R2 refer to the first and second rounds of spraying during the year. Data sources: CARE India IRS coverage surveys; Kala-azar Management Information System (KAMIS), National Vector Borne Disease Control Program (NVBDCP).

Until 2015, DDT was the only insecticide used by the Indian VL control program. Evidence of DDT resistance has been mounting in India for decades (Mukhopadhyay et al., 1990; Dhiman et al., 2003; Dinesh et al., 2010; Singh and Kumar, 2015). In one recent study conducted in three VL endemic states in India, sand fly mortality rates ranged from 31 to 89% (Singh et al., 2012). Beginning in 2015, the national program began to shift from DDT to a synthetic pyrethroid (SP), alphacypermethrin (5%) (Coleman et al., 2015). In 2015, both SP and DDT were used; by 2016 all IRS utilized SP (**Figure 1**).

WHO guidelines recommend two spray rounds per year, targeted to the two annual peaks of sand fly density (WHO, 2010). This recommendation has been further refined to time the rounds based on seasonal vector density data gathered by the VL control program. Currently, timed to precede peak vector densities, the first round of spraying is usually scheduled from March to April, and the second round from August to September. As of 2017, IRS is recommended inside all human dwelling rooms, inside cattle sheds and walls of open verandahs to cover all the rooms used for sleeping. In India, spraying is targeted to villages that have reported at least one VL case in the previous 3 years.

As part of the VL elimination program, a series of quality improvements have been implemented or are planned. Modern hand compression pumps (HCP) were introduced instead of conventional stirrup pumps (Coleman et al., 2015; Kumar et al., 2020). A systematic IRS monitoring program was initiated in 2014 to provide IRS household coverage data via regular sample surveys. Estimated household coverage in targeted villages was 51% in 2014 and increased rapidly to 72% in 2015 and >80% in all subsequent years (CARE-India, 2020). SP was discontinued in 2018 when insecticide quality was found to be below standards, and was reintroduced in limited areas for the first round of 2019, and in all areas from the second round of 2019 onwards. For 2020, the first round of SP-based IRS in Bihar state was conducted during April–May where 5,842 villages were sprayed in 33 districts. However, delays have resulted from the COVID-19 pandemic in 2020. The pandemic has also affected health services and had an adverse impact on case detection and thus the reported VL incidence. These disruptions add to the challenges of interpreting the impact of IRS on VL incidence.

No formal large-scale evaluation of the impact of IRS on VL incidence has ever been conducted in India, and data for IRS program impact on entomological parameters show mixed results. Some focal studies demonstrated a reduction in sand fly populations 15 days after SP-IRS that was more pronounced than with earlier DDT-based IRS (Kumar et al., 2020). Other, more extensive evaluations failed to find substantial reductions in sand fly density in sprayed compared to unsprayed villages (Poche et al., 2018a). In surveillance data from Bihar, 82% of the reduction in VL incidence from the peak of 2007 to 2018 was achieved by 2013, when IRS coverage was patchy and did not exceed 40% of targeted households. Only 18% of the total reduction in incidence was seen during 2013 to 2018, when IRS coverage more than doubled to reach >80% of targeted households.

Other interventions, most crucially reservoir reduction through earlier diagnosis and more effective treatment of both VL and post-kala-azar dermal leishmaniasis (PKDL), were implemented over the same period of time (Singh et al., 2016). Notable changes included provision of rapid diagnostic tests and newer treatment drugs at the primary health clinic level. SSG was phased out and replaced by miltefosine between 2008 and 2012; miltefosine was replaced by single dose liposomal amphotericin B for VL treatment by the end of 2015 (**Figure 1**). After this date, miltefosine was reserved for PKDL treatment. Moreover, systematic active case detection was implemented starting in 2017, which substantially reduced the time from illness onset to diagnosis and treatment (CARE-India, 2020).

Thus, current data leave a large degree of uncertainty regarding the effectiveness of IRS as the sole vector control strategy. An evidence-based strategy directed at long-term control of VL cases and sand fly populations is needed to improve the likelihood that VL incidence can be maintained at the current low levels going forward.

### Insecticide Treated Bed Nets

**Figure 2:**
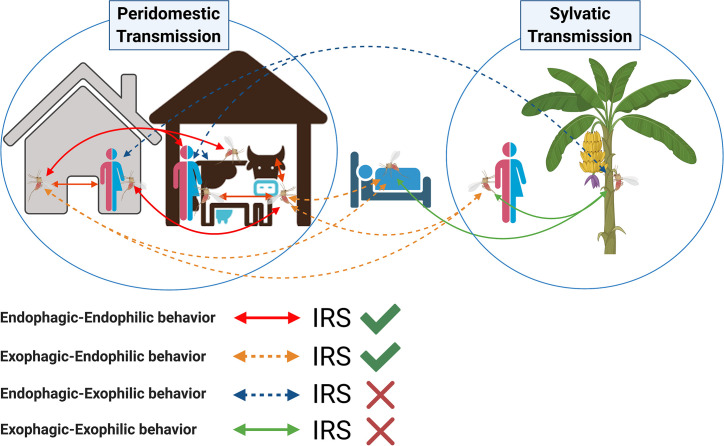
Potential role of indoor residual spraying (IRS) in controlling transmission of visceral leishmaniasis by *Phlebotomus argentipes* sand flies. Arrows indicate all possible scenarios of visceral leishmaniasis transmission taking into consideration the change in *Ph. argentipes* behavior from endophilic and endophagic to exophilic and exophagic. The accepted peridomestic transmission cycle assumes that *Phlebotomus argentipes* has an endophagic-endophilic behavior (red-solid lines); in this scenario indoor residual spraying is effective (green tick). Sand flies with an exophagic–endophilic behavior (orange-dotted lines) may rest inside the walls of IRS-sprayed houses and cattle sheds after taking a blood meal outdoors; in this scenario IRS is also effective. Other suspected scenarios of sylvatic transmission include a change in *Ph. argentipes* behavior from endophilic to exophilic, where a sand fly would rest outdoors after taking a blood meal indoors (endophagic–exophilic:blue-dotted lines), or outdoors (exophagic–exophilic:green-solid lines); in these two scenarios IRS is not effective (red cross).

Insecticide treated nets (ITNs) combine the individual protection of a bed net with the effect of an insecticide, and represent another vector control measure to complement IRS (Joshi et al., 2009). Long-lasting insecticide nets (LLINs) are a class of ITNs with the insecticide incorporated into the material of the net by the manufacturer. ITNs, including LLINs, have been shown to be effective against malaria and cutaneous leishmaniasis in other parts of the world (Wilson et al., 2014). However, in the only controlled trial of LLINs in the ISC, no significant impact on reduction of either VL incidence or vector density was demonstrated. The KalaNet trial was a cluster randomized controlled trial of LLINs conducted in India and Nepal (Picado et al., 2010b). Only a 25% reduction in *Ph. argentipes* density was reported for houses in intervention villages compared to controls (Picado et al., 2010a). More importantly, there was no difference in the rate of seroconversion over 24 months in residents of intervention villages compared to those in control villages, suggesting that LLINs were not sufficient to decrease the incidence of infection in these communities. The authors hypothesized that outdoor transmission might explain this negative result (Picado et al., 2010b). In contrast, a study conducted in Bangladesh reported a 66.5% reduction in VL incidence after insecticide impregnation of existing bed nets already in use by village inhabitants (Mondal et al., 2013). Potential explanations for these discrepancies include differences in study design and human behavior, and the possibility that, due to DDT application in the face of falling susceptibility, *Ph. argentipes* in India had become more exophilic and exophagic (**Figure 2**) than the same species in Bangladesh, where no systematic IRS occurred prior to 2012. In fact, sand fly abundance in traps placed in vegetation and the outskirts of endemic villages support the contention that *Ph. argentipes* in Bihar are now exophilic (Poche et al., 2018b).

Other factors that may influence ITN effectiveness include adherence to net use and sleeping location. Adherence was not directly measured in either study. In a study in one of the most endemic districts of Bihar, 95% of residents were said to sleep outside during the warm months of the year (Perry et al., 2013). By contrast, in an epidemiological study in the most highly endemic sub-district of Bangladesh, fewer than 5% of participants reported ever sleeping outdoors (Bern et al., 2005). These factors may have influenced the observed differences but have not been directly compared. Sleeping outdoors, however, does not preclude the use of ITNs but may require certain adaptations to promote their use, exemplified in some distribution programs in Sudan (Ritmeijer et al., 2007). These data suggest that adaptation of the net and the bed frame may improve ITN usage.

### Environmental Modification

Environmental modification (EVM) comprises changes to the home or surrounding environment by covering or filling in cracks and crevices in walls and floors of the houses where sand flies rest. Dwellings in VL-affected areas of India are frequently made of mud, thatch and brick with or without plaster, and have mud/earthen, brick, or cement floors (Perry et al., 2013; Malaviya et al., 2014). Cracks and crevices are prevalent in these structures and provide dark, humid refuges for sand flies. Palpable dampness in the earthen floors of such houses has also been shown to be associated with VL risk (Bern et al., 2010). Most local houses are suited to habitation by *Ph. argentipes*, but thatched homes with mud floors contain a higher number of sand flies (Malaviya et al., 2014).

When compared to IRS and ITNs, data are limited for EVM as a VL control modality. A study conducted in 15 homes in an endemic village of Bihar used a mud and lime plaster mix to seal cracks in homes and cattle enclosures reducing sand fly numbers from 11.5 to 2.0 per man hour collections within a month of sealing (Kumar et al., 1995). The study by Joshi et al. conducted to compare the efficacy of IRS, ITNs and EVM in India, Nepal, and Bangladesh also found an average decline in sand fly abundance of 42% for all study sites observed over five months after the walls of homes and cattle enclosures were plastered with a mixture of mud and lime (Joshi et al., 2009). Interestingly, another study in Bangladesh found no significant difference in sand fly densities in treated compared to control sites when crevices were filled with mud alone (Chowdhury et al., 2011a). It was hypothesized that lime decreases sand fly populations due to high pH or moisture-limiting properties, potentially inhibiting indoor breeding of *Ph. argentipes* (Kumar et al., 1995; Joshi et al., 2009). In Vaishali district, Bihar, EVM by cement plastering of the walls of 300 houses from two villages showed a 60.2% reduction in sand fly density when compared to the control village (Dinesh et al., 2017).

The 2009 report by Joshi et al. is the only study which compared village-wide use of IRS, LLINs, and EVM in India, Nepal, and Bangladesh (Joshi et al., 2009). Overall, IRS, LLINs, and EVM reduced sand fly density by 72.4, 42.0, and 43.7%, respectively, for at least 5 months irrespective of wall materials or presence of cattle in the house. IRS was effective for sand fly reduction in all three sites, whereas LLINs were only effective in Bangladesh and India. Notably, in India, the LLIN results in this study were contrary to those of the KalaNet trial conducted around the same time period (Picado et al., 2010a), and IRS was effective despite utilizing DDT. Lime plastering resulted in a significant reduction of sand fly numbers in India and one of two Nepali study sites (Joshi et al., 2009). This study was conducted under research conditions in which incentives were provided for participation in plastering, and where community helpers visited weekly for inspection and households treated with IRS were asked not to re-plaster their walls. As such, some of these approaches may not be applicable on a large scale by national vector control programs. EVM, IRS, and ITN generally target endophilic populations of sand flies. Populations that are exophilic and reside in outdoor enclosures and vegetation would remain a risk factor for VL (**Figure 2**).

## Knowledge Gaps Underpinning Failures in Vector Control Efforts in Bihar, India

### Vector Biology of *Ph. argentipes*

Recently, major gaps have been exposed in our understanding of *Ph. argentipes* sand fly bionomics and behavior that have important implications for effectiveness of vector control tools (Cameron et al., 2016). *Ph. argentipes* has traditionally been considered a poor flier that mainly feeds and rests indoors, a main factor in favoring IRS for the elimination of VL in the ISC (Chowdhury et al., 2016). However, a series of studies of Bihari villages have demonstrated that *Ph. argentipes* is more exophilic and exophagic than previously believed (Poche et al., 2011; Poche et al., 2012; Poche DM et al., 2017; Poche et al., 2018b). *Ph. argentipes* sand flies, including blood fed females, were found in higher numbers in peri-domestic and semi-sylvatic vegetation niches such as banana plants and female palmyra palm trees than in cattle sheds and dwellings and were collected from tree canopies at 18.4 m high, contradicting the conventional belief that they remain within 6 ft of the ground (Poche et al., 2011; Poche et al., 2012; Poche DM et al., 2017; Poche et al., 2018b). The high numbers of female sand flies consistently captured in banana plants and female palmyra palm trees may be due to the sugar-rich sap they produce (Poche et al., 2012; Poche DM et al., 2017; Poche et al., 2018b). Moreover, despite the dogma that adult sand flies seldom travel more than 100 m from their breeding site (Dinesh et al., 2009), adult females travelled further than males and were found up to 300 m from their breeding sites (Poche et al., 2011; Poche et al., 2012; Poche et al., 2018b). Of note, a recent study found that *Ph. sergenti*, *Ph. mongolensis*, *Ph. orientalis* and *Lutzomyia longipalpis*, major vectors of leishmaniasis from the Old and New World, preferentially feed on *Cannabis sativa* plants (Abbasi et al., 2018). *Cannabis sativa* is abundant in VL endemic villages in Bihar and yet its importance as a sugar source for *Ph. argentipes* has not been investigated to date. Importantly, in these Bihari villages, a high proportion of *Ph. argentipes* were found to have fed on humans (41%), followed by cattle (20%), and a mix of human–cow (17%), and human–buffalo (5%) blood (Garlapati et al., 2012). Similarly, a large scale-study involving 24 villages from Bihar state found that the majority of *Ph. argentipes* sand flies fed on human (81%) and/or bovine blood (60%) (Poche et al., 2018a). Altogether, these studies establish that *Ph. argentipes* sand flies readily feed on humans and may be exophilic and even exophagic. Clearly, these characteristics would diminish the efficacy of interventions that target only endophilic sand fly populations (**Figure 2**). Vector control tools targeting both exophilic and endophilic sand fly populations are required, and there is a need for in-depth studies to fully understand what factors account for this shift in behavior, and the extent to which it is specific to Bihar (Cameron et al., 2016). In addition, fundamental entomological investigations designed to establish the longevity, feeding preferences, oviposition sites, and resting behavior of *Ph. argentipes* are urgently indicated to inform an evidence-based strategy for effective long-term vector control.

### Reservoirs of *L. donovani*

In anthroponotic VL, humans form the infection reservoir. PDKL, in particular, is considered the major interepidemic reservoir because such patients can have skin lesions for years without being systemically ill (Zijlstra et al., 2017). Recent studies of kala-azar and PKDL patients confirm historical data on their infectiousness (Molina et al., 2017; Mondal et al., 2019; Singh et al., 2021). Overall, 55.3% of enrolled PKDL patients had a positive direct xenodiagnosis result after sand flies fed on patients’ lesions; the proportion was highest for those with nodular lesions but macular PKDL patients also infected sand flies at a substantial rate (Mondal et al., 2019). A mathematical model that fitted data from CARE India and KalaNet studies of endemic districts in Bihar, India, predicted that the VL elimination campaign threshold would be met for India by 2020, but that low-level disease transmission will still continue (Le Rutte et al., 2017). The model assumes that, in addition to VL and PKDL patients, asymptomatic carriers play a role in VL transmission, though with a much lower rate of infectiousness (Le Rutte et al., 2017). Studies that provide quantitative data needed to establish the potential of asymptomatic individuals as reservoirs for parasite transmission to vector populations are in progress (Singh et al., 2014; Tiwary et al., 2017; Singh et al., 2021). Non-human reservoir hosts have been sought but never proven to play a role in leishmanial transmission in the ISC (Singh et al., 2020). The paucity of definitive studies adds an extra challenge for the elimination program and highlights the need to understand all potential scenarios of disease transmission, particularly as VL incidence has fallen to unprecedentedly low levels (Cameron et al., 2016; Singh et al., 2020).

### Human Behavior

Human behavior affects the likelihood of exposure to sand flies (**Figure 2**). In India, sleeping outdoors in the warm months is extremely common, reported at 95% in one study (Perry et al., 2013) and 50.1% in another (Govil et al., 2018). Some people (usually adult men or boys) may sleep in the cattle shed with their animals, potentially increasing sand fly exposure (Singh et al., 2010a; Poche et al., 2011). However, the effect of cattle on VL risk may be mixed, since their presence may increase vector density but attract feeding sand flies away from humans (Bern et al., 2010). Another behavioral habit that may be relevant to vector-transmission of *L. donovani* by *Ph. argentipes* in India is open defecation, which was associated with significantly elevated risk of VL in a recent investigation in Bihar (Priyamvada et al., 2021). Despite government campaigns to change it, open defecation is still common in India, and represents one of the highest rates in the world. In 2015–2016, the Demographic and Health Survey estimated that 39% of Indians practice open defecation (National Family Health Survey, 2017). Importantly, it is frequently practiced in peridomestic vegetation at dawn and dusk when sand flies are most active (Dinesh et al., 2009). Such behavior may also provide organic matter suitable for sand fly breeding and could promote a semi-sylvatic transmission cycle which would remain untouched by IRS.

## Innovative Tools to Sustain Vector Control After Achieving the Elimination Target

Better integrated vector control strategies and alternative tools are clearly needed to sustain the elimination target threshold and to control VL outbreaks.

### Biomarkers of Vector Exposure

One of the major limitations to the evaluation of vector control programs is having accurate estimates of the rates of human exposure to sand fly bites to assess risk of disease transmission. Currently, surveillance tools to estimate sand fly prevalence in endemic areas are limited to indoor sand fly catches by aspiration or light traps (Dinesh et al., 2008). However, these entomological methods do not directly evaluate whether implemented vector control strategies reduce human exposure to vector bites.

During a blood meal, sand flies deposit a repertoire of salivary proteins into the host. Humans living in endemic areas are constantly exposed to sand fly bites and elicit a strong antibody response specific to sand fly salivary proteins (Andrade and Teixeira, 2012). Importantly, multiple studies have proposed sand fly salivary proteins as biomarkers of human exposure to the bite of several *Leishmania* vectors (Clements et al., 2010; Souza et al., 2010; Gidwani et al., 2011; Marzouki et al., 2011; Marzouki et al., 2015; Mondragon-Shem et al., 2015; Carvalho et al., 2017; Sumova et al., 2018).

The idea of a marker of exposure to *Ph. argentipes* bites was first evaluated against total *Ph. argentipes* saliva pre- and post-intervention in a cohort of people living in Bihar, India, and in control households of the KalaNet trial in Nepal (Clements et al., 2010; Gidwani et al., 2011). These studies revealed that *Ph. argentipes* saliva contained immunogenic proteins that induce a robust antibody response. Furthermore, anti-saliva antibodies dropped 30 days after VL patients were maintained in a hospital under supervised conditions, with no sand fly exposure, then increased above baseline 6 months after leaving the hospital, likely due to re-exposure to sand fly bites (Clements et al., 2010). Similar observations by Gidwani et al. showed a drop of anti-saliva antibodies at 12 and 24 months post-intervention when compared to baseline values (Gidwani et al., 2011). These studies suggest that persistence of antibodies to *Ph. argentipes* saliva without further exposure is limited, and reinforces the usefulness of this tool to monitor changes in vector-human contact over time. However, significant cross-reactivity was observed against saliva of *Ph. papatasi*, the other human-biting sand fly species found in the area (Clements et al., 2010; Gidwani et al., 2011). In addition, substantial variability between preparations, and labor constraints, limit the usefulness of total saliva for large-scale field applications. Finding immunodominant and species-specific recombinant sand fly salivary proteins can overcome these limitations. Recombinant salivary proteins from other sand fly species have been validated as good biomarkers to assess human-vector exposure in leishmaniasis endemic areas (Souza et al., 2010; Teixeira et al., 2010; Marzouki et al., 2012; Marzouki et al., 2015; Carvalho et al., 2017; Sumova et al., 2018). Identification of appropriate recombinant salivary proteins that function as specific markers of exposure to *Ph. argentipes* will facilitate reliable long-term assessment of the intensity of the human-vector contact over time during the post-VL elimination era and how it might correlate with the occurrence of disease outbreaks. More studies are also needed to assess the persistence of *Ph. argentipes* salivary antibodies under various conditions. A recent study demonstrated the absence of a humoral response to sand fly saliva in cold climate leishmaniasis-endemic areas with short sand fly seasons (Oliveira et al., 2020). This indicates that antibodies against vector saliva can be transient, and that their durability is likely to be dependent on the level of exposure and length of the sand fly season. As such, it will be informative for vector control programs to study the fluctuation in levels of anti-saliva antibodies throughout different seasons of *L. donovani* transmission in India.

### Biological Control of *Ph. argentipes*

Biological alternatives to insecticide spraying have been explored for the control of vector-borne pathogens, either by suppressing vector populations or by eliminating their capability to transmit pathogens. Studies have recently linked gut microbiota to survival of *Leishmania* in the sand fly midgut (Kelly et al., 2017; Louradour et al., 2017). Moreover, mature infections with *Leishmania* significantly reduced the diversity of gut microbiota favoring the persistence of some bacterial families over others (Kelly et al., 2017; Louradour et al., 2017). This opens up the possibility that elimination of distinct microbiota positively associated with parasite growth and development may adversely affect vector competence. Another study identified *Bacillus megaterium* and *Brevibacterium linens* as non-pathogenic commensals in midgut microbiota of field-collected *Ph. argentipes* (Hillesland et al., 2008). The authors proposed a para-transgenic approach to adversely affect vector competence of *Ph. argentipes* by transforming these bacteria to express anti-leishmania molecules (Hillesland et al., 2008). Further, *Wolbachia*, an intracellular symbiont bacterium that has been previously used for effective control of mosquitoes (Moreira et al., 2009; Walker et al., 2011; Caragata et al., 2016; Dutra et al., 2016; O’Neill, 2018; Pereira et al., 2018), has been recently reported from sand flies (Da Silva Goncalves et al., 2019; Vivero et al., 2019). In-depth knowledge of the composition of gut microbiota from field-collected *Ph. argentipes* sand flies, how it varies by biotope and, most importantly, after infection by *L. donovani*, is needed. Such studies would shed light on the feasibility of designing new strategies and tools to prevent disease transmission by manipulating vector competence.

### Other Proposed Tools for Vector Control

Poche et al., published a series of papers on the effectiveness of treating cattle with a systemic insecticide, fipronil, as an effective tool for control of sand fly populations (Poche et al., 2013; Poche et al., 2020). In a controlled study, 100% mortality was observed in both adult sand flies and larvae that fed on cattle treated orally with one dose of 4 mg fipronil/kg, or their feces, respectively (Poche et al., 2013). The same group then used a series of simulations and showed that if 50% of sand flies fed on cattle treated with fipronil over 12 months, the intervention would be 52–62% effective in reducing sand fly numbers (Poche et al., 2020). This probability increased to 89–97% if the model also assumed that more than 50% of sand flies also oviposited in feces of treated cattle (Poche et al., 2020). Though promising, the use of fipronil in cattle needs to be tested in a large-scale field study, as it was only tested on a small number of animals per group in a controlled environment (Poche et al., 2013). Moreover, potential opposition by household members to frequent administration of fipronil to their cattle may pose an additional barrier and limitation to the wide application of this tool in the field. An additional concern regards the potential for contamination of milk used for human consumption. This aspect needs to be thoroughly addressed prior to implementation of this control strategy. Worth noting, this tool has been shown to be effective against mosquitoes, so it may be possible to target both vectors simultaneously (Fankhauser et al., 2015; Poche et al., 2015; Poche RM et al., 2017).

Genetic strategies such as CRISPR-Cas have also been proposed for manipulation of sand fly vectors, and could be potentially applied for the generation and release of sterile males in the field (Jeffries et al., 2018; Martin-Martin et al., 2018; Louradour et al., 2019). However, low survival rates of embryos after manipulation of eggs or adult females present challenges to the use of this technology in sand flies.

## Conclusion

Efforts to fill our knowledge gaps in vector biology are necessary. Ideally, studies should integrate clinical data with entomological data in longitudinal large-scale studies to establish the true nature of *L. donovani* transmission; unfortunately, studies frequently focus on one or the other. Moreover, entomological studies should assess the density of *Ph. argentipes* sand flies in nearby peri-domestic and semi-sylvatic vegetation as well as in houses and cattle sheds to accurately assess the effect of interventions on both endophilic and exophilic sand fly populations. Additionally, geographic spatial analysis of the distribution of VL cases, and structured questionnaires for inhabitants of endemic villages, where sleeping and other social and behavioral activities are taken into consideration, are necessary to complement the entomological data. This would address important questions regarding the importance of outdoor populations of *Ph. argentipes* in transmission of *L. donovani*, their contribution to indoor populations, and their interaction with humans.

The initial assumptions on which the VL elimination program is based need to be revisited. This includes the use of IRS that solely targets indoor sand fly populations as the only implemented vector control tool. Additionally, studies addressing all possible transmission cycle scenarios and the human behavior that supports them will begin to overcome what is at present a major limitation to the success of the elimination campaign.

## Author Contributions

RG, EI, TS, JV, SS, CB, and SK conceived and wrote the manuscript. PM and BR managed IRS coverage assessment, data analysis, and interpretation. BS provided project insight, data analysis, and interpretation. All authors contributed to the article and approved the submitted version.

## Funding

This work was supported by the Bill & Melinda Gates Foundation, Grant Number (INV-008856/OPP1196454), http://www.gatesfoundation.org/ and by the Intramural Research Program of the NIH, National Institute of Allergy and Infectious Diseases. The authors are solely responsible for the content of this manuscript.

## Conflict of Interest

The authors declare that the research was conducted in the absence of any commercial or financial relationships that could be construed as a potential conflict of interest.
